# An Information Theoretic Approach to Symbolic Learning in Synthetic Languages

**DOI:** 10.3390/e24020259

**Published:** 2022-02-10

**Authors:** Andrew D. Back, Janet Wiles

**Affiliations:** School of Information Technology and Electrical Engineering, The University of Queensland, Brisbane, QLD 4072, Australia; j.wiles@uq.edu.au

**Keywords:** information theoretic models, synthetic language, entropy, Zipf–Mandelbrot–Li law, language models, behavior prediction

## Abstract

An important aspect of using entropy-based models and proposed “synthetic languages”, is the seemingly simple task of knowing how to identify the probabilistic symbols. If the system has discrete features, then this task may be trivial; however, for observed analog behaviors described by continuous values, this raises the question of how we should determine such symbols. This task of symbolization extends the concept of scalar and vector quantization to consider explicit linguistic properties. Unlike previous quantization algorithms where the aim is primarily data compression and fidelity, the goal in this case is to produce a symbolic output sequence which incorporates some linguistic properties and hence is useful in forming language-based models. Hence, in this paper, we present methods for symbolization which take into account such properties in the form of probabilistic constraints. In particular, we propose new symbolization algorithms which constrain the symbols to have a Zipf–Mandelbrot–Li distribution which approximates the behavior of language elements. We introduce a novel constrained EM algorithm which is shown to effectively learn to produce symbols which approximate a Zipfian distribution. We demonstrate the efficacy of the proposed approaches on some examples using real world data in different tasks, including the translation of animal behavior into a possible human language understandable equivalent.

## 1. Introduction

Language is the primary way in which humans function intelligently in the world. Without language, it is almost inconceivable that we as a species could survive. Language is clearly far richer than mere words on a page or even spoken words. Almost every observable dynamic system in the world can be considered as having its own language of “words” that carry meaning. Put simply, we propose that “every system is language”.

In contrast to classical value-based models such as those employed in signal processing, or even the concept of quantized models employing discrete values such as those found in classifiers, we propose that the next phase of AI systems may be based on the concept of *synthetic languages*. Such languages, we suggest, will provide the basis for AI to learn to interact with the world in varying levels of complexity, but without the technical challenges of infinite expressions [1] and building natural language processing systems based on human language.

The concept of synthetic languages is that it is possible to derive a model to capture meaning which is either emergent or assigned in natural systems. While this approach can be understood for animal communications, we suggest that it may be useful in a wide range of other domains. For example, instead of modeling systems based on hard classifications based on some measured features, a synthetic language approach could be useful for developing an understanding of meaning using behavioral models based on sequences of probabilistic events. These events might be captured as simple language elements.

This approach of synthetic language stems from earlier work we have done based on entropy-based models. Normally, entropy requires a large number of samples to estimate accurately [2]. However, we have previously developed an efficient algorithm which permits entropy to be estimated with a small number of samples which has enabled the development of simple entropy-based behavioral models. For example, an early application has shown promising results, successfully detecting dementia in a control group of patients by listening to their conversation using a simple synthetic language consisting of 10 “synthetic words” derived from the inter-speech pause lengths [3].

The basis for this approach is the view that probabilistically framed behavioral events derived from dynamical systems may be viewed as words within a synthetic language. In contrast to human languages, we hypothesize the existence of synthetic languages defined by small alphabet sizes, limited vocabulary, reduced linguistic complexity and simplified meaning.

Yet, how can a systematic approach be developed which determines such synthetic words? Usually the aim of human speech recognition is to form a probabilistic model which enables short term bursts of speech audio to be classified as particular words. However, with the approach being proposed here, we might consider meta-languages where other speech elements, perhaps sighs, coughs, emotive elements, coded speech, specific articulations or almost any behavioral phenomena can be considered as a synthetic language.

The challenge is that we are seeking to discover language elements from some input signals, yet while we can know the ground truth of human language elements and hence determine the veracity of any particular symbolization algorithm, this task is not straightforward for synthetic languages where we do not have access to a ground truth dataset.

Hence, while much of computational natural language processing relies significantly on modeling complex probabilistic interactions between language elements resulting in models such as hidden Markov models, and smoothing algorithms to capture the richness and complexities of human language, in our synthetic language approach the aims are considerably lower. However, even with the significantly reduced complexity, we have found significant potential.

Consider one of the most basic questions of language—what are the language elements? Language can be viewed as observing one or more discrete random variables *X* of a sequence *X* = $X_{1},\ldots ,X_{i},\ldots ,X_{K}$ $,X_{i}=x\in X^{M},$ that is, $x_{i}$ may take on one of *M* distinct values, $X^{M}$ is a set from which the members of the sequence are drawn, and hence $x_{i}$ is in this sense symbolic, where each value occurs with probability $p\left(x_{i}\right),$ i∈[1,M]. One of the earliest methods of characterizing the probabilistic properties of symbolic sequences was proposed by Shannon [4,5] who proposed the concept of entropy, which can be defined as

(1) $$H_{0}\left(X\right)=-\sum_{i=1}^{M}p\left(x_{i}\right)\log_{2}\left(p\left(x_{i}\right)\right)$$

Entropy methods have been applied to a wide range of applications, including language description [6,7,8], recognition tasks [9,10,11,12,13], identification of disease markers through human gene mapping [14,15], phylogenetic diversity measurement [16], population biology [17] and drug discovery [18].

An important task in applying entropy methods and subsequently in deriving synthetic languages is the seemingly simple task of knowing what the probabilistic events are. If the system has discrete features, then this task may be trivial; however, for continuous-valued analog observed behaviors, this raises the question of how we should determine what the symbols are. For example, suppose we wish to convert the movement of a human body into a synthetic language. How can the movements, gestures or even speech be converted into appropriate symbols?

This symbolization task is related to the well known problem of scalar quantization [19,20] and vector quantization [21,22] where the idea is to map a sequence of continuous or discrete values to a symbolic digital sequence for the purpose of digital communications. Usually the aim of this approach is data compression so that the minimum storage or bandwidth is required to transmit a given message within some fidelity constraints. Within this context, a range of algorithms have been derived to provide quantization properties.

Earlier work using vector quantization has been proposed in conjunction with speech recognition and speaker identification. A method of speaker identification was proposed using a k-means algorithm to extract speech features and then vector quantization was applied to the extracted features [23].

An example of learning a vector quantized latent representation with phonemic output from human speech was demonstrated in [24]. The concept of combining vector quantization with extraction of speaker-invariant linguistic features appears in applications of voice conversion, where the aim is to convert the voice of a source speaker to that of a target speaker without altering the linguistic content [25]. A typical approach here is to performing spectral conversion between speakers, such as using a Gaussian mixture model (GMM) of the joint probability density of source and target features or by identifying a phonemic model to isolate some linguistic features and then perform a conversion, while minimizing some error metric such as the frame by frame minimum mean square error.

A common aspect of these models is generally to isolate some linguistic features and then perform to vector quantization. Extensions of this work include learning vector quantization [26], with more recent extensions to deep learning [27], federated learning [28], entropy-based constraints [29]. A neural autoencoder incorporating vector quantization was demonstrated to learn phonemic representations [30]. A method for improving compression on deep features using an entropy-optimized loss function for vector quantization and entropy coding modules to jointly minimize the total coding cost was proposed in [31].

In the work we present here, our interest is rather different in its goals from vector quantization and hence the algorithms we derive take a different direction. In particular, while vector quantization approaches tend to be aimed at efficient communications with minimum bit rates, we seek to discover algorithms which might uncover emergent language primitives. For example, given a stream of continuous values, it is possible to find a set of language elements which might be understood as letters or words. While it may be expected that such language based coding representations will also provide a degree of compression efficiency, this is not necessarily our primary goal.

The goal of symbolization can therefore be differentiated from quantization in that the properties of determining language primitives may be very different from simply efficient data compression or even fidelity of reconstruction. For example, these properties may include metrics of robustness, intelligibility, identifiability, and learnability. In other words, the properties, goals and functional aspects of language elements are considerably more complex in nature than those used in vector quantization methods.

In this paper, we propose an information theoretic approach for learning the symbols or letters within a synthetic language without any prior information. We demonstrate the efficacy of the proposed approaches on some examples using real world data in quite different tasks including the translation of the movement of a biological agent into a potential human language equivalent.

## 2. Synthetic Language Symbols

### 2.1. Aspects of Symbolization

Consider a sequence of continuous or discrete valued inputs. How can we obtain a corresponding sequence of symbols derived from this input? An *M*-level quantizer is a function which maps the input into one of a range of values. A quantizer is said to be optimum in the Lloyd-Max sense if it minimizes the average distortion for a fixed number of levels *M* [19,32].

A symbolization algorithm converts any continuous-valued random variable input u(t) to a discrete random variable *X* of a sequence *X* = $X_{1},\ldots ,X_{i},\ldots ,X_{K},$ where $X_{i}=x\in X^{M}.$ In this sense, $x_{i}$ can be viewed as a component in an alphabet or finite nonempty set with symbolic members and dimensionality M.

Hence, a trivial example of symbolization is the *M*-level quantization by direct partitioning of the input space. In this case, for some continuous random variable set u(t) then we have an output $\left\{x(t)\right\}$ where

(2) $$x\left(t\right)=X_{i}:U_{i}\leq u\left(t\right)\leq U_{i+1}\;\forall i,i=0,\ldots ,M$$

where the quantization levels $\left\{U_{i}\right\}$ are bounded as $U_{0}=\inf\left(u(t)\right),U_{M}=\sup\left(u(t)\right),$ and where $\left\{U_{i}\right\}$ are chosen according to any desired strategy, for example $U_{i}<U_{i+1}$ ∀i, and $U_{i}∼\Phi \left(\mu ,\sigma \right).$ This approach partitions the input space such that the greater the value of the input the higher the symbolic code in the alphabet and where the partitions are distributed according to a cumulative normal distribution. This simple probabilistic algorithm encompasses the full input space, and has been demonstrated to provide useful results.

However, while any arbitrary symbolization scheme can be applied, in the context of extracting language elements it is reasonable to derive methods with preservation properties. Previously we have derived entropy estimation algorithms which include linguistic properties such a orthographic constraints with Zipf–Mandelbrot–Li distribution [33]. Here we consider symbolization using a similar concept of preserving linguistic properties. In the first instance, we consider a symbolization method which introduces a Zipf–Mandelbrot–Li constraint, ensuring the derived language symbols reflect Zipfian properties. We then introduce a constraint which seeks to preserve a defined language intelligibility properties. This approach can be further extended to consider properties such as preservation of prediction.

Another approach we propose is to systematically partition the input space according to linguistic probabilistic principles so that the derived language approximates natural language. A method of doing this to partition the input space such that each region has an associated probability which approximates the expected natural language events.

The methods we present here can be compared to prior work such as universal algorithms for classification and prediction [34]. In contrast to these prior approaches however, there are some major differences. In particular, since our interest is in language, the sequences we consider are non-ergodic [35,36]. However, universal classification methods are usually associated with ergodic processes [37]. Hence, these differences lead us to consider a very different approach than these prior works.

While not only technically different, this means language symbolization requires very different approaches than those developed for ergodic universal classifiers. Our aim in this paper is to present and explore some of these different approaches which we consider further below.

### 2.2. Zipf–Mandelbrot–Li Symbolization

Given the task of symbolization, we seek to constrain the output symbols to properties which might reflect some of the linguistic structure of the observed sequence. There are clearly many ways in which this can be achieved and in this algorithm we propose a simple approach of probabilistic constraint as a first step in this direction.

For natural language, described in terms of a sequence of probabilistic events, it has been shown that Zipf’s law [38,39,40,41,42,43] describes the probability of information events that can generally be ranked into monotonically decreasing order. This raises the question of whether it possible to derive a symbolization algorithm which produces symbols which follow a Zipfian law. The idea here is that since we are seeking an algorithm which will symbolize the input to reflect language characteristics, then it is plausible that such an algorithm will produce symbols which will approximate a Zipfian distribution. In this section, we propose such a symbolization method.

We have previously proposed a new variation of the Zipf–Mandelbrot–Li (ZML) law [2,39,44], which models the frequency rank *r* of a language element $x\in \Sigma^{M+1}$ from an alphabet of size M+1. An advantage of this model is that it provides a discrete analytic form with reasonable accuracy given only the rank and the alphabet size. Moreover, it has the further advantage that it can be extended to include linguistic properties to improve the accuracy when compared against actual language data [33].

Hence, a symbolization process based on the ZML law can be obtained and a derivation for this symbolization algorithm based on the original development in [39,45,46] is shown in Appendix A. Using this approach, where a precise value of the ranked probability is available or each rank, given alphabet size M, then given a partitioned input space, the algorithm firstly enables the ordering of each partition in terms of its probabilistic rank such that each partition corresponds the closest ZML probability. We then derive an algorithm termed CDF-ZML to constrain the partition probabilities with probabilistic characteristics approximating a ZML distribution.

**Figure 1:**
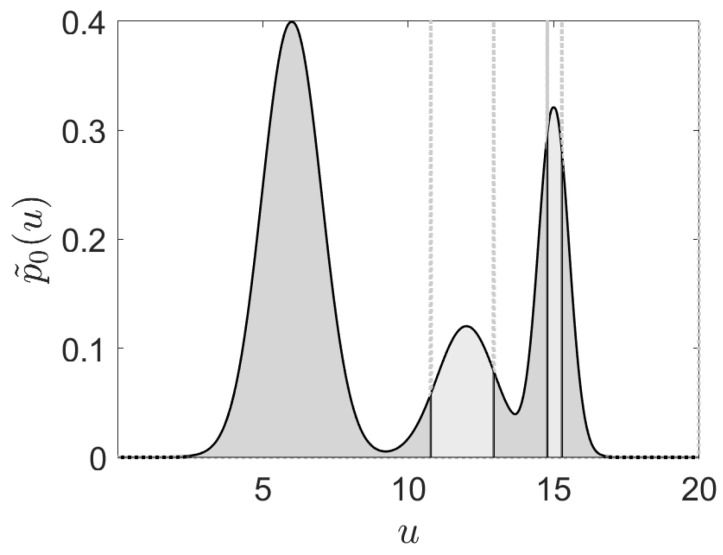
The proposed CDF-ZML linguistic symbolization algorithm partitions the input space to provide symbols which follow a Zipfian distribution. In this one dimensional example, the symbols with highest rank are left-most and are followed by successively smaller probabilities as indicated by the area under the curve. The symbols are defined by the occurrence of a continuous valued input within a given partition. Hence each symbol has a corresponding probability defined by a Zipfian–Mandelbrot–Li distribution.

An example of the CDF-ZML approach with the resulting partitioning is shown in Figure 1, where it can be observed that the most probable symbol occurs initially, and is then followed by successively smaller probabilities (indicated by the area under the curve).

In contrast to simple *M*-level quantization schemes, this approach to partitioning is not designed to optimize some metric of data compression, but rather it is a nonlinear partitioning of the input space to ensure the probability distribution of the data will follow an approximately Zipfian law. Thus, it may be viewed as a first step in seeking to symbolize an input sequence as language elements. Note that we cannot claim that the elements do indeed form the basis for actual language elements, so this is to be regarded as a first, but useful step. In human language terms, this could be potentially useful for example, in the segmentation of audio speech signals into phonemic symbols [47]. Hence, it may be similarly useful in applications of synthetic language to extract realistic language elements.

Clearly this approach can be further extended, however, to consider other language-based symbolization methods. In the next section we consider another methodology which examines a more sophisticated property of language and how they may be incorporated into symbolization processes.

### 2.3. Maximum Intelligibility Symbolization

Intelligibility is an important communications property which has received considerable interest [48,49,50]. Hence, a symbolization method which can potentially enhance intelligibility may be useful for deriving language based symbols.

Are there any precedents of naturally occurring intelligibility maximization? In other words, since we are interested in synthetic languages which are not restricted to human language, are there other examples of naturally occurring forms of language or information transmission systems which seek to enhance a measure of intelligibility as a function of event probabilities? In fact there are such examples which occur in natural biological processes.

Intelligibility optimization is observed in genetic recombination events during meiosis where interference which reduces the probability of recombination events is a function of chromosomal distance [51]. In this case, genes closer together encounter greater interference, and hence undergo fewer crossing over events, while for genes that are far apart crossover and non-crossover events will occur in equal frequency. Another way to view this is that the greatest intelligibility of genetic crossing over occurs with distant genes.

Accordingly, in this section, we develop a symbolization algorithm which seeks to optimize intelligibility. Note that there are various metrics for defining intelligibility, and so the approach we propose here is not intended to be optimal in the widest sense possible, other approaches are undoubtedly possible and may yield better results or be better suited to particular tasks or languages.

Based on the concept that probabilistically similar events could be confused with each other, especially in the presence of noise, intelligibility can be evidently defined as a function of the distance between probabilistically similar events. In this case, the idea is that the symbols which are closest in probabilistic ranking should be made as distant as possible in input space. Hence, we can introduce a probability-weighted constraint $\lambda_{c}$ to be maximized such as

(3) $$\lambda_{c}=\sum_{\tau ,\pi_{r}}\psi_{\pi }{\left(\tau -\pi_{r}\right)}^{2}$$

where $\psi_{\pi }$ enables a continuously variable weighting to be applied to the probabilistic lexicon of symbolic events with symbols with distance indices τ and $\pi_{r}.$

This approach can be generalized in various ways to include optimization constraints for features such as robustness, alphabet size or variance of the symbolic input range. Hence, if we require a symbolization scheme which will provide improved performance in the presence of noise, then it may be useful to consider constraints which take this into account.

Here we propose a symbolization approach using a probabilistic divergence measure as an intelligibility metric. The goal is that the symbolization algorithm will produce a sequence of symbols which have a property of maximum intelligibility. The way we do this is to ensure that nearby symbols are the least likely to occur together. This is somewhat similar to the principle used in the typewriter QWERTY keyboard, where the idea was to place keys together which are unlikely to be used in succession [52].

The proposed *MaxIntel* algorithm is aimed to producing a symbolization scheme which arranges nearby symbol partitions, according to the value of the input, to maximize probabilistic intelligibility. The derivation of the symbolization algorithm with maximum intelligibility constraints is given in Appendix B.

**Figure 2:**
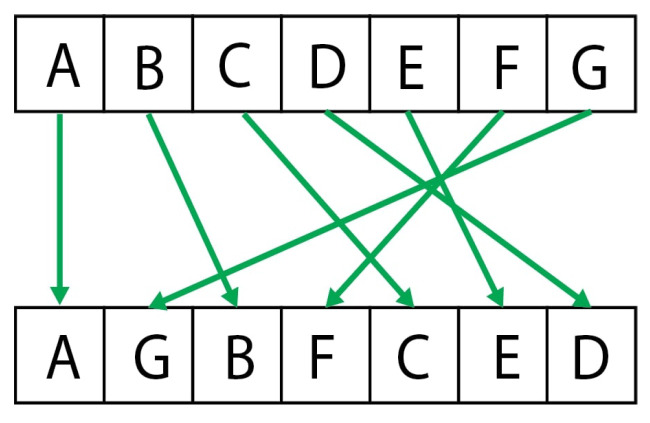
A natural extension of the symbolization process is to optimize the intelligibility of the observed sequence. The rank ordering process of the proposed intelligibility symbolization algorithm is illustrated here.

A diagram of this intelligibility CDF indexing is shown in Figure 2.

**Figure 3:**
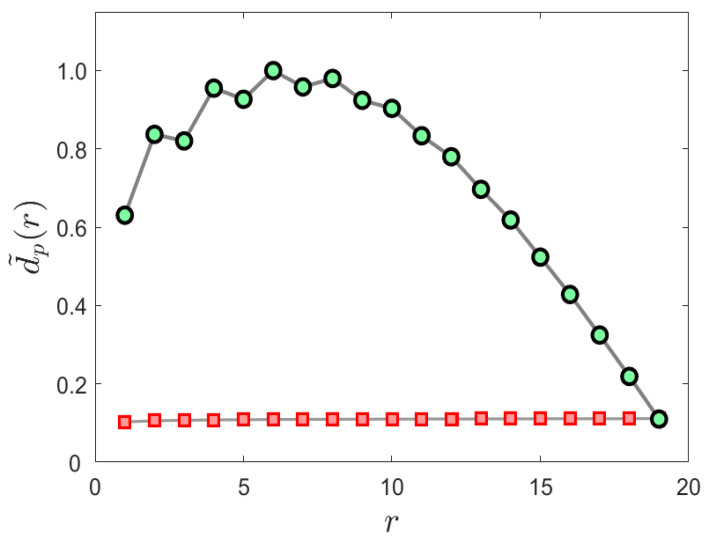
The performance of the proposed maximum intelligibility symbolization algorithm is shown in this example. The lower red curve shows the intelligibility of regular symbolization, while the upper green curve shows the intelligibility of the proposed algorithm as a function of probabilistic symbol rank. In this case the alphabet size is *M* = 20.

The performance of the proposed intelligibility constrained symbolization algorithm is shown in Figure 3. In this case, partitioning is constrained to maximize a probabilistic divergence measure between sequential unigrams which optimizes the probabilistically framed intelligibility.

## 3. Learning Synthetic Language Symbols

### A Linguistic Constrained EM Symbolization Algorithm (LCEM)

In contrast to the constructive symbolization algorithms considered in the previous section, here we propose an adaptive neural-style learning symbolization algorithm suitable for symbolizing dynamical systems where the data are provided sequentially over time. In particular, we present an adaptive algorithm which constrains the symbols to have a linguistically framed probability distribution.

The approach we propose is a symbolization method based on the well known Expectation-Maximization (EM) algorithm [53]. However, a problem with regular EM symbolization is that there is no consideration given to language constraints which might provide a more realistic symbolization with potential advantages.

Hence, we derive a probabilistically constrained EM algorithm which seeks to provide synthetic primitives which conform to a Zipfian–Mandelbrot–Li distribution corresponding to the expected distribution properties of a language alphabet. The derivation of the Linguistic Constrained Expectation-Maximization (LCEM) algorithm is given in Appendix C.

The way in which the LCEM algorithm operates is that a set of the clusters are initialized and then an entropic error is minimized progressively as a probabilistic constraint by adapting the mean and variance of each cluster. In this manner, a new weighting for each cluster is obtained which is a function of the likelihood and the entropic error. This continues until the cluster probabilities converge as indicated by the entropy or other some other criterion has been met, such as time to converge.

**Figure 4:**
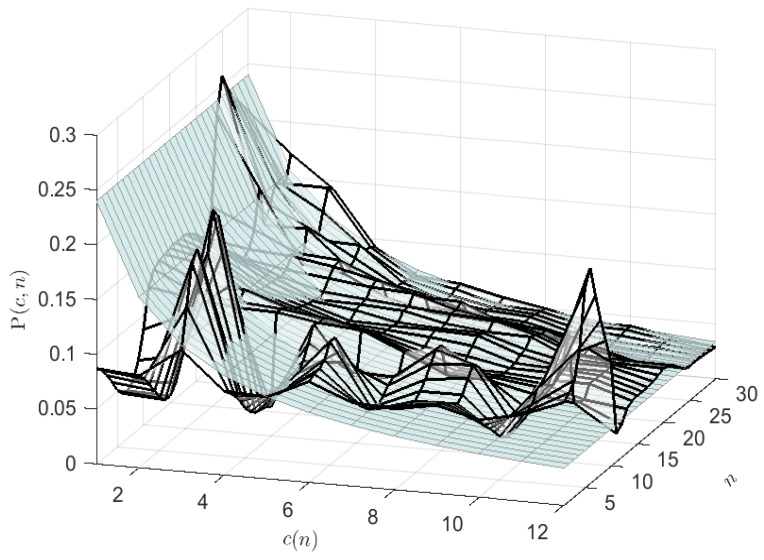
The performance of the LCEM adaptive linguistic symbolization algorithm is shown. In this case, the probabilistic surface of the clusters are shown as they adapt. After 30 steps, the convergence of the cluster probabilities (meshed surface) can be observed to converge to the ZML probability surface (shaded).

The convergence performance of the LCEM symbolization algorithm is shown in Figure 4. In this case, the probabilistic surface of the clusters are shown during the adaptive learning process. The convergence of the symbol probabilities can be observed to converge to the ZML probability surface.

## 4. Example Results

### 4.1. Authorship Classification

In this section, we present some examples demonstrating the application of symbolization. It should be noted that our intention is not to validate symbolization using simulations, rather we simply present some potential applications which show that useful results can be obtained. We leave it to the reader to explore the potential of the presented methods further. The examples also show that symbolization by itself does not necessarily solve a task, but it can be an important part of the overall approach in discovering potential meaning when applied to real world systems. Hence, we can expect that symbolization will be only part of a much more comprehensive model.

In this example, we pose the problem of detecting changing authorship of a novel without any pretraining. This is not intended to be a difficult challenge; however, it is included to demonstrate the concept of using symbols within an entropy-based model to determine some characteristics of a system based on a sequence of input symbols.

For the purpose of the example, the text was filtered to remove all punctuation and extra white space, and hence $X^{M}=\left[a,b,\ldots ,z,sp\right].$ The text was then symbolized with M=5 based on the word-length with $\left\{s_{0}\left(n_{k}\right)\right\}:n_{1}<3,4\leq n_{2}<6,7\leq n_{3}<9,10\leq n_{4}<12,13\leq n_{5}.$

In this case, the initial dataset consisted of the text from “*The Adventures of Sherlock Holmes*”, which is a collection of twelve short stories by Arthur Conan Doyle, published in 1892. This main text was interspersed with short segments from a classic children’s story, “*Green Eggs and Ham*” by Dr. Seuss published in 1960. The full texts were symbolized and the entropy was estimated using the efficient algorithm described in [33], applied to non-overlapping windows of 500 symbols in length.

The way in which authors are detected is then based on measuring the short-term entropy of the input text features. We do not necessarily know in advance what the characteristics of the text will be; hence, simplified methods of measuring average word lengths are not so helpful. Hence, in this case, by assigning a symbol to different word length ranges, the idea is that the entropy will characterize the probabilistic distribution of the input features.

To detect the different authors, we can introduce a simple classification applied to the entropy measurement. In this case, we use the standard deviation of entropy, but for a higher dimensional example, a more sophisticated classification scheme could be used, for example, a k-means classifier.

**Figure 5:**
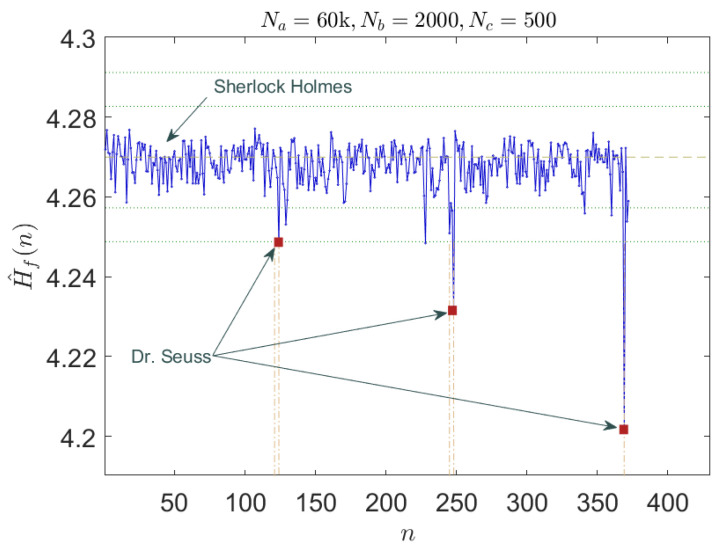
The proposed symbolization algorithm used in conjunction with a fast entropy estimation model readily detects different authors as shown here. An entire novel is represented across the horizontal axis.

The results are shown in Figure 5 where it is evident that the different authors are clearly identifiable in each instance by a significant drop in entropy when the different author is detected. Clearly this simple demonstration could be extended to multiple features using more complex classifiers; however, we do not do this here.

### 4.2. Symbol Learning Using an LCEM Algorithm

The behavior of the proposed LCEM algorithm is considered in this section. A convenient application to examine is a finite mixture model where the cluster probabilities are constrained towards a ZML model. In this case, we consider a small synthetic alphabet which has a set of M=12 symbols.

**Figure 6:**
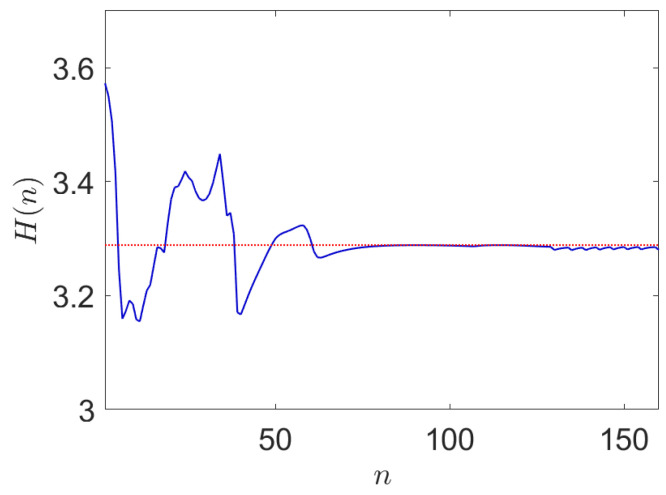
The entropy convergence behavior of the proposed linguistic constrained EM algorithm. Here an example is shown of how the LCEM algorithm performs on a sample multivariate data set for M=12.

The performance of the LCEM algorithm when applied to a sample multivariate dataset for M=12 is shown in Figure 6.

Hence, the proposed LCEM algorithm is evidently successful in deriving a set of synthetic symbols from a multivariate dataset with unknown distributions by adapting a multivariate finite mixture model. It can be observed that the convergence performance of the proposed LCEM algorithm occurs within a small number of samples. Interestingly, since the optimization is based on the likelihood but constrained against the entropic error, the gradient surface is nonlinear, and hence we observe an irregular, non-smooth error curve.

**Figure 7:**
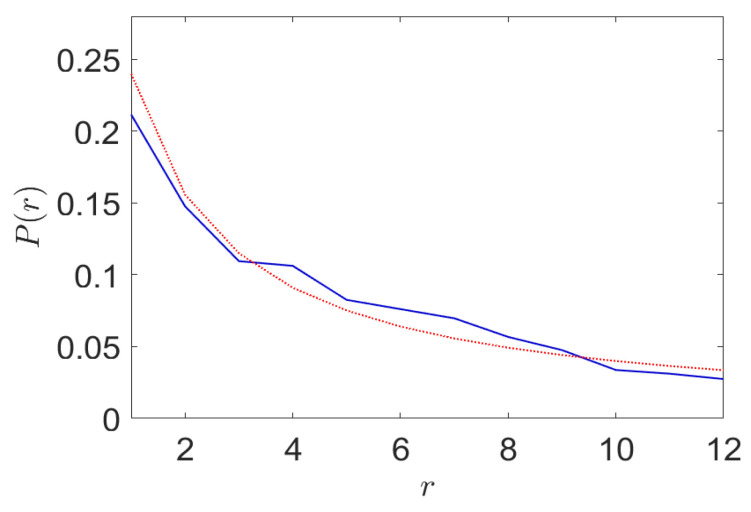
An example of the LCEM algorithm performance in adapting the cluster probabilities (solid line) as compared to the theoretical ZML distribution (dotted line) when near convergence.

It is of interest to examine the convergence of the cluster probabilities and an example of the LCEM algorithm performance is shown in Figure 7 where the cluster probabilities are compared to the theoretical ZML distribution.

### 4.3. Potential Translation of Animal Behavior into Human Language

This example is presented as a curious investigation into the potential applications of symbolization and synthetic language. Our interest is in discovering ways of modeling and understanding behavior using these methods, and so we certainly do not claim that this is a definitive method of animal behavior into a human language form. However, we found it somewhat interesting, even if speculative in the latter stage, and hence it is intended to stimulate discussion and ideas rather than provide a definitive solution to this task.

**Figure 8:**
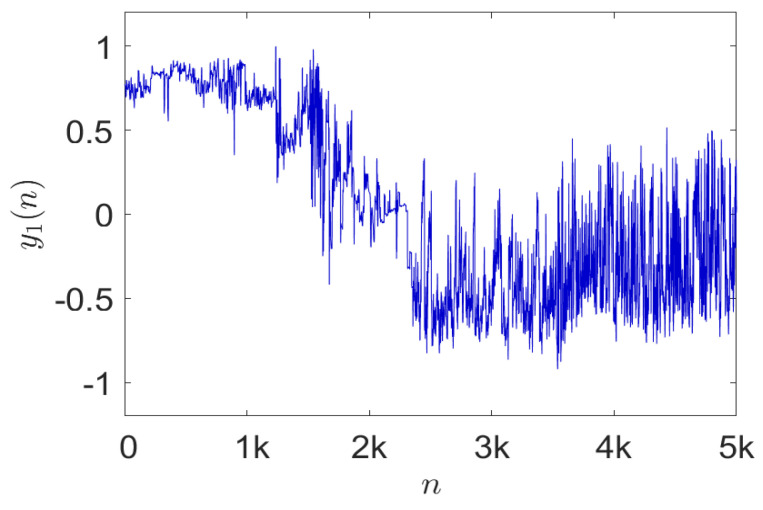
Behavioral time series data obtained from a triaxial accelerometer attached to a wild Kalahari meerkat recorded over about 3 min and sampled at 100 Hz.

In part, our application is motivated by the highly useful data collected by Chakravarty [54] from triaxial accelerometers attached to wild Kalahari meerkats. The raw time series data from one of the sensors over a time period of about 3 min are shown in Figure 8.

Analysis of animal behavior has received considerable attention and recent work based on an information theoretic approach includes an entropy analysis of behavior of mice and monkeys using a two types of behavior [55]. A range of information theoretic methods including relative entropy, mutual information and Kolmogorov complexity were used to analyze the movements of various animals using binned trajectory data in [56]. An analysis of observed speed distributions of Pacific bluefin tuna was conducted using relative entropy in [57]. The positional data between pairs of zebra fish were analyzed using transfer entropy to model their social interactions in [58].

It is evident that information theoretic approach can yield a deeper understanding of animal behavior. A common aspect of these and other prior works that we are aware of, are that they are restricted to using entropy-based methods. In our case, we are considering the possibility of extending this approach further by treating the symbols as elements within a synthetic language. Hence, we are interested to raise the question of whether it is possible to obtain an understanding of biological or other behaviors using a synthetic language approach. To the best of our knowledge this approach has not been considered previously in this way. Hence, this will be demonstrative and exploratory in nature, rather than a definitive analysis.

The first step in our example, is to symbolize the observed data. In contrast to most entropy based techniques, a larger alphabet size of symbols can be readily accommodated within the analysis; however, for the purpose of this case, we select a smaller number of samples which ensures the input range is fully covered.

**Figure 9:**
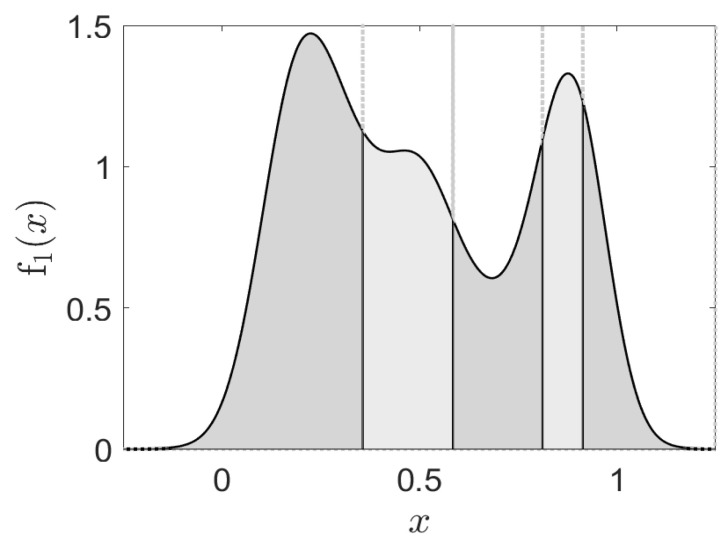
Applying the CDF-ZML symbolization algorithm to the meerkat behavioral data provided this symbolic pdf which is then used to symbolize the data.

The CDF-ZML symbolization algorithm with a synthetic alphabet size of M=5 was applied to 10 s of meerkat behavioral data. A ZML distribution was generated according to (A1)–(A9). The symbolic pdf output is shown in Figure 9.

Hence, the raw meerkat behavioral data are symbolized, where for convenience in this example, we use letters to represent each symbol. Note that we could also consider a richer set of input data as symbols. For example, using n-grams or frequency domain transformed inputs, or a combination of these methods.

Observing a synthetic language requires determining letters, spaces and words. Hence, symbolization provides the first stage in discovering the equivalent of letters in the observed sequence. The next step is to determine spaces which in turn enables the discovery of words. However, even this simple task is not necessarily so trivial.

In human language, it is generally found that the most frequent symbol is a space. Following a similar approach, we found that it is useful to determine which symbol or symbols separate words in the symbol sequence. In human speech or written language, a space or pause is simply represented by a period of silence. However, in behavioral dynamics, particularly when there is nearly constant movement, such a period of “silence” does not necessarily have a corresponding behavioral characteristic of no movement.

In this context, we consider that the functional role of a space is essentially a “do-nothing” operation. Hence, in an animal species which displays almost constant movement, there are effectively six types of behaviors which we propose can constitute a functional space. These correspond to forward and reverse directions in each of the three axes. The actual identification of these functional spaces is then found by measuring the movements in each of these directions which occur with the highest frequency. Note that this does not mean that the animal is actually doing nothing. It means that in terms of an information theoretic perspective, the symbols have the highest relative probability of occurring and therefore convey little “surprising” information.

**Figure 10:**
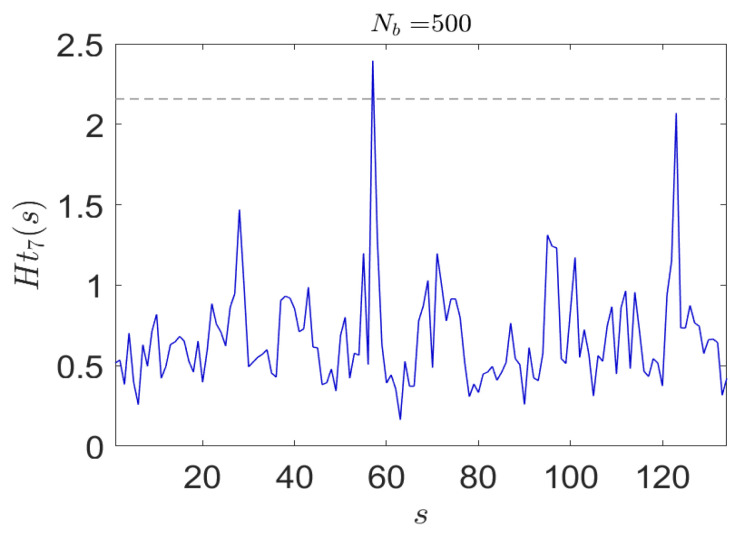
Entropy of the Kalahari meerkat movement when symbolized using the proposed CDF-ZML algorithm. The quasi-periodic time series reveals structure in terms of low and high frequency periodic behavior corresponding to the predictably frequent occurrence of words with different probabilities.

As a first step, the entropy of the resulting symbolic sequence can be computed and is shown in Figure 10. This reveals some structure in terms of low and high frequency periodic behavior. Such periodic probabilistic behavior is to be expected in natural languages since it may correspond to random, but predictably frequent words with different probabilities [59].

**Figure 11:**
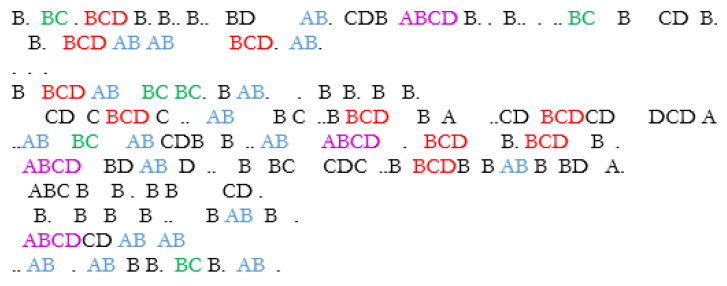
Symbolic output from the Kalahari meerkat movement data obtained using CDF-ZML symbolization algorithm. The symbols are represented as letters and the synthetic words are clearly evident.

A symbolic sequence obtained from the meerkat raw sensory data are shown in Figure 11. For convenience, the symbols are represented by letters which enables the visualization of the synthetic language words. In this case, the synthetic language spaces are replaced by regular spaces, which enables the synthetic language words to be viewed.

When viewing this sequence of symbols perhaps the first question that can be asked is “how can this be understood”? The well known *direct method* is one approach for determining the meaning of the words within languages [60]. This method relies on a number of aspects; however, it principally requires some form of direct matching of real world objects or tasks and the related words. Consequently this causes some disadvantages including the difficulty of learning and the time required.

Grammar translation is another approach for translating between languages and is based on knowledge of the rules, grammar and meaning of words in both languages [61]. However, for the task of learning the meaning of a new synthetic language for which we do not have an understanding of the language itself, this method is not likely to be useful.

One possible approach which may be useful for translating synthetic language is to consider methods based of understanding the functional aspects of the language. The idea of communicative-functional translation is to view translation as related to communication between specific actors [62]. Hence, we suggest that a probabilistic functional translation method might be of interest to consider in this case.

How can such probabilistic functionality be measured and adopted in such a potential translation application? It is clearly not possible to use the probabilistic structure across a large vocabulary, and universal structure is notoriously uncommon across languages [63]. However, we suggest that it may be possible to consider an approach of cross-lingual transfer learning based on probabilistic structure of parts of speech (POS) [64,65].

It is evident that despite the existence of a large number of POS across various languages, and some disagreement about the definitions, there is strong evidence that a set of coarse POS lexical categories exists across all languages in one form or another [66]. This indicates that while fine-grained relative cross-lingual POS probabilities may vary, when linked to the same observed linguistic instantiations, the cross-lingual probabilities of coarse POS categories are likely to be similar [67,68,69]. Therefore, while we have used probabilistic POS rankings from an English language corpora in this example, since we are using only coarse-grained categories, this means that the rankings might be expected to be stable across languages.

There is some closeness of ranked probabilities between some POS categories and this presents some degree of uncertainty in the results. One approach to consider this further in future work would be to seek to introduce functional grammatical structure based on probabilistic mappings. That is, in the present example we are only considering very simple probabilistic rankings of coarse POS; however, a future approach might extend the concept of probabilistic rankings to more complex linguistic properties as employed in current POS tagging systems to disambiguate POS and reduce the uncertainty of the many possible English-language translations [70,71].

**Figure 12:**
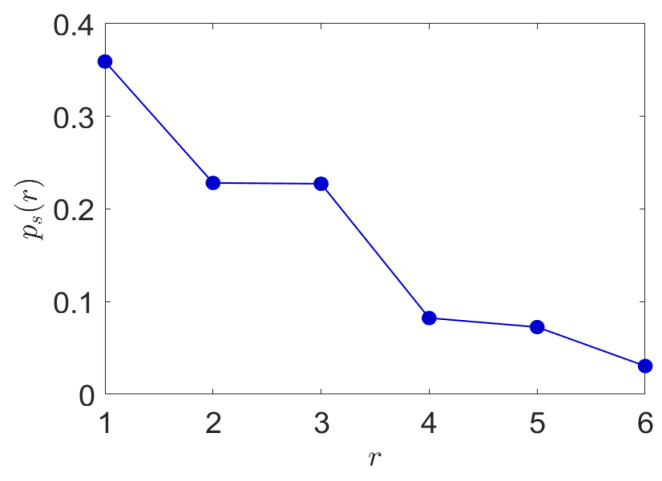
Normalized ranked probabilistic characteristics of parts of speech from the Brown Corpus. This is used as a first step in our proposed translation approach based on cross-lingual transfer learning based on probabilistic parts of speech.

**Figure 13:**
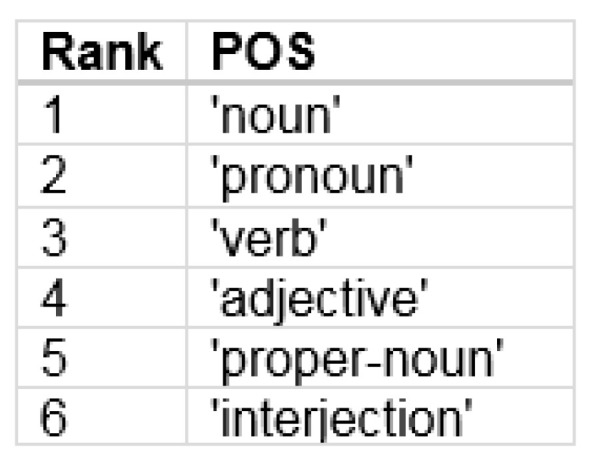
The specific parts of speech obtained from the experimentally determined ranked probabilities of parts of speech from a range of corpora.

Hence, as a next step in this investigation, we determined the probabilistic characteristics of coarse-grained POS observed in English using the Brown Corpus. The normalized ranked probabilities are shown in Figure 12 and the specific types of speech are shown in Figure 13.

**Figure 14:**
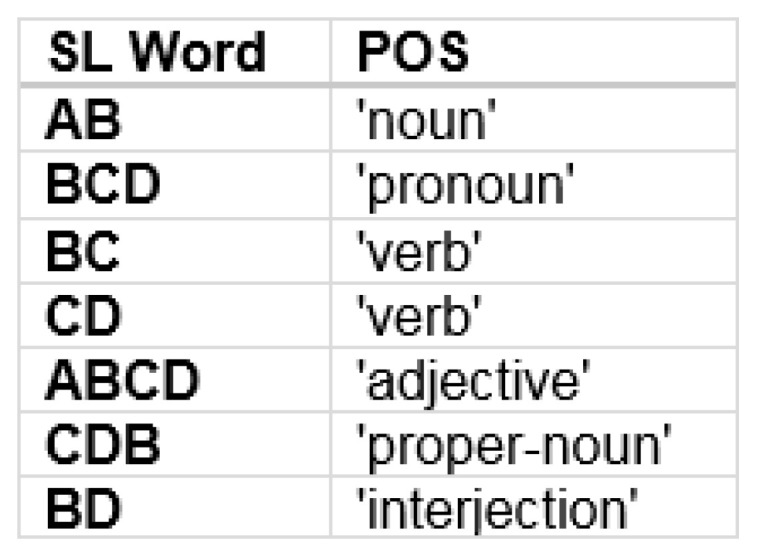
The probabilistic rankings of the English language coarse syntactic parts of speech are used to map the synthetic language words with the same rankings.

The synthetic words can be ranked according to probabilities and because the vocabulary is limited, we map these to corresponding parts of speech in human language with the same relative probabilistic rankings. This would not necessarily be easily done for large vocabularies, but because the synthetic language under consideration has a small vocabulary, we can readily form the mapping as shown in Figure 14. This approach assumes that there exists the same POS in both languages which correspond according to probabilistic ranking. However this appears to be a reasonable assumption when considering sets of coarse syntactic POS categories which omit finer-grained lexical categories as we do in this example [66,70,71,72].

While the cross-lingual POS mapping gives some possible insight into the potential meaning of the observed synthetic language, we thought it would be of interest to view a form of potential high probability words corresponding to the sequence of observed POS. Hence, we proposed the concept of visualizing what the sequence would potentially “look like” if it were written in a human understandable form. Therefore the next step is not intended to provide an accurate translation of the actual behavior, but rather as a means of trying to view one possible narrative which could fit the observations.

**Figure 15:**
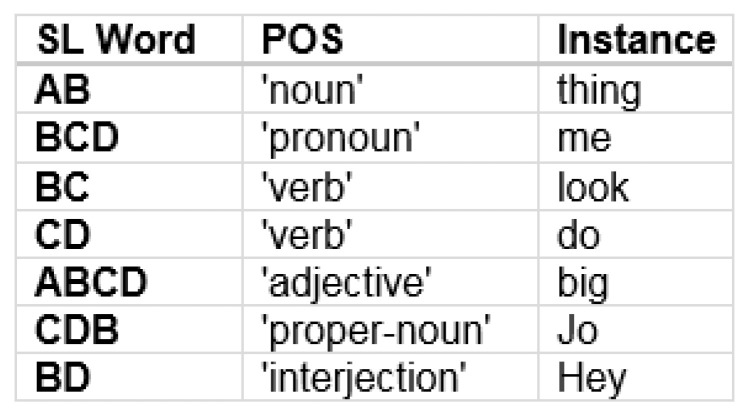
The synthetic language words can be mapped to potential parts of speech according to their relative probabilities. Then a potential human language translation can be formed by associating place-holder words with the synthetic language “words” corresponding to specific parts of speech.

Accordingly, we analyzed the Brown corpus to obtain the most frequent words corresponding to each of the coarse syntactic POS categories. We then assigned each of these most frequent words to the categories as shown in Figure 15. We do not claim that these words are what is actually “spoken” by the behavior, but provides a novel way of seeking to view a potential narrative for this example.

In this way, instead of viewing the synthetic words such as “BCD”, “AB”, etc., we first map them to a coarse level syntactic POS. From this point, the POS are mapped to recognizable human language words. Although admittedly speculative, this last stage provides an interesting insight into how we might begin to understand the possible meaning of unknown synthetic language texts.

**Figure 16:**
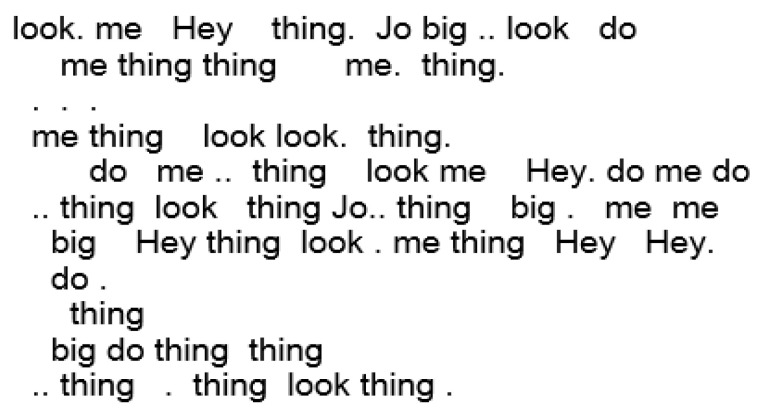
The synthetic language of the meerkat behavior is first translated into coarse syntactic POS categories which omit finer-grained lexical categories. Then, using the most frequent probabilistically ranked POS words as place-holders, we can then provide a possible, but speculative narrative.

The resulting output in human language is shown in Figure 16, where a simplistic, but recognizable synthetic dialog can be seen emerging.

Interestingly, although not shown in the results here, the behavior of the meerkat can be viewed as doing nothing of interest for a period of time—almost like an extended period of “silence” and then followed by a period of high activity. It is in this high activity time that we show the output “translation” results. These last stage results are included as a curiosity.

A next step from this point is to explore ways of ascertaining more reliable word translations. For example, embedding ground truth by direct learning is one possibility, though with distinct disadvantages as noted earlier. However, another more promising approach appears to be extending the concept of communicative functional grammar, which we can consider in terms of conditional probabilistic structures. However, these topics are beyond the scope of this paper and will be explored in the future.

## 5. Conclusions

Many real world systems can be modeled by symbolic sequences which can be analyzed in terms of entropy or synthetic language. Synthetic languages are defined in terms of symbolic primitives based on probabilistic behavioral events. These events can be viewed as symbolic primitives such as letters, words and spaces within a synthetic language characterized by small alphabet sizes, limited word lengths and vocabulary, and reduced linguistic complexity.

The process of symbolization in the context of language extends the concept of scalar and vector quantization from data compression and fidelity to consider explicit linguistic properties such as probabilistic distributions and intelligibility.

In contrast to human languages, where we know the language elements including letters, words, functional grammars and meaning, for synthetic languages, even determining what constitutes a letter or word is not trivial. In this paper we propose algorithms for symbolization which take into account such linguistic properties. We propose a symbolization algorithm which constrains the symbols to have a Zipf–Mandelbrot–Li distribution which approximates the behavior of language elements forms the basis of some linguistic properties.

A significant property for language is effective communication across a medium with noise. Hence, we propose a symbolization method which optimizes a measure of intelligibility. We further introduce a linguistic constrained EM algorithm which is shown to effectively learn to produce symbols which approximate a Zipfian distribution.

We demonstrate the efficacy of the proposed approaches on some examples using real world data in different tasks, including authorship classification and an application of the linguistic constrained EM algorithm. Finally we consider a novel model of synthetic language translation based on communicative-functional translation with probabilistic syntactic parts of speech. In this case, we analyze behavioral data recorded from Kalahari meerkats using the symbolization methods proposed in the paper. Although not intended to provide an accurate or authentic translation, this example was used to demonstrate a possible approach to translating unknown data in terms of synthetic language into a human understandable narrative.

The main contributions of this work were to introduce new symbolization algorithms which extend earlier quantization approaches to include linguistic properties. This is a necessary step for using entropy based methods or synthetic language approaches when the input data are continuous and no apparent symbols exists in the measured data. We presented various examples of using the symbolization algorithms to real world data which demonstrate how it may be effectively applied to analyzing such systems.

## Data Availability

The Brown corpus used in Section 4 is available as part of the NLTK from https://www.nltk.org/book/ch02.html (accessed on 18 August 2021). Data used for the meerkat analysis were derived from data deposited in the Dryad Digital Repository https://doi-org.ezproxy.library.uq.edu.au/10.5061/dryad.7q294p8 (accessed on 18 August 2021) (Chakravarty et al., 2019).

## Appendix A. Derivation of Zipf–Mandelbrot–Li Probabilistic Symbolization Algorithm

The approach we consider here is to derive an algorithm which partitions an input space, which may be continuously valued, such that for some input sequence, the output will be constrained to a particular probabilistic distribution. The constraint we use is a well-known Zipfian distribution which is frequently observed in language primitives.

In previous work we have proposed a new variation of the Zipf–Mandelbrot–Li (ZML) law [2,39,44]. The advantage of this model is that it provides a discrete analytic form with reasonable accuracy given only the rank and the alphabet size. Moreover, it has the further advantage that it can be extended to include linguistic properties to improve the accuracy when compared against actual language data [33].

The symbolization process based on the ZML law can be derived as follows. Firstly we describe the formulation of the ZML law. For any random word of length L, given by $v_{k}\left(L\right)=\left\{w_{s},x_{1},\ldots ,x_{L},w_{s}\right\},\;k=1,\ldots ,M^{L}$ the frequency of occurrence is determined as

(A1) $$p_{i}\left(L\right)=\frac{\lambda }{{\left(M+1\right)}^{L+2}}\;i=1,\ldots ,M^{L}$$

where the model uses the frequency rank *r* of a language element $x\in \Sigma^{M+1}$ from an alphabet of size M+1. Li showed that λ can be determined as [39]:

(A2) $$\begin{array}{cc} \sum_{L=1}^{\infty }M^{L}p_{i}\left(L\right) & =1 \end{array}$$

(A3) $$\begin{array}{cc} \sum_{L=1}^{\infty }M^{L}\frac{\lambda }{{\left(M+1\right)}^{L+2}} & =\frac{\lambda M}{{\left(M+1\right)}^{2}}\;\; \end{array}$$

(A4) $$\begin{array}{cc} \lambda & =\frac{{\left(M+1\right)}^{2}}{M} \end{array}$$

which leads to

(A5) $$p_{i}\left(L\right)=\frac{1}{M{\left(M+1\right)}^{L}}\;i=1,\ldots ,M^{L}$$

Now, defining the rank of a given word $v_{k}\left(L\right)$ as r(L), the probability of occurrence of a given word in terms of rank can be defined as [45,46]:

(A6) $$p\left(r\right)=\frac{\gamma }{{\left(r+\beta \right)}^{\alpha }}$$

This allows the model to be determined as $\gamma^{\prime }=\gamma /\kappa ,$ $p\left(r\right)=\gamma^{\prime }/{\left(r+\beta \right)}^{\alpha },$ where the model parameters can be found as [39]:

(A7) $$\begin{array}{cc} \alpha & =\frac{\log_{2}\left(M+1\right)}{\log_{2}\left(M\right)}\\ \beta & =\frac{M}{M+1}\\ \gamma & =\frac{M^{\alpha -1}}{{\left(M-1\right)}^{\alpha }} \end{array}$$

Hence, the probabilities of the distribution can be determined as

(A8) $$p\left(r\right)=\frac{M^{\alpha -1}}{{\left[\left(M-1\right)\left(r+\beta \right)\right]}^{\alpha }}$$

where

(A9) $$\sum_{i=1}^{M}p\left(i\right)=1,\;\sum_{i=1}^{\infty }\frac{\gamma }{{\left(r+\beta \right)}^{\alpha }}=\kappa$$

This means that for a given alphabet size M, and for each rank, a precise value of the ranked probability is available. This will be useful in our discrete probability mass estimate for each event in a symbolic alphabet as shown below.

Given a partitioned input space, the first step we can take is to nominate the probabilistic rank of each partition. While the depiction in Figure 1 shows a partitioning strategy which has the highest rank left-most, the actual partitioning could be in any rank order.

Hence, we require a strategy for selecting the indices of the partition that correspond the closest ZML probability. Therefore, we define the set of required ZML indices as $\left\{\pi_{r}\right\}$ r=0,…,M and $\left\{{\hat{p}}_{U}\left(U_{i}\right)\right\}$ as the initial set of probabilities associated with the partitioned input space, and where $\left\{p_{z}\left(r\right)\right\}$ is the set of probabilities according to (A7)–(A9). Then we can obtain the required set of indices according to the simple criteria

(A10) $$\pi_{rk}=\left\{\begin{array}{cc} 1 & \mathrm{if}\;k=\underset{i}{arg min}\left(\left|p_{z}\left(r\right)-{\hat{p}}_{U}\left(U_{i}\right)\right|\right)\\ 0 & \mathrm{otherwise} \end{array}\right.$$

where k=i such that $\left|p_{z}\left(r\right)-{\hat{p}}_{U}\left(U_{i}\right)\right|$ is minimized among all values of ${\hat{p}}_{U}\left(U_{i}\right),$ for a given $p_{z}\left(r\right)$.

Hence $\pi_{rk}$ can be found by a simple search procedure in $O\left(n^{2}\right)$ time to determine the set of partition ranges with probabilities $\left\{{\hat{p}}_{U}\left(\pi_{r}\right)\right\}$ that most closely corresponds to the ZML model $\left\{p_{z}\left(r\right)\right\}$ defined by (A7)–(A9).

Clearly, while this is a useful step, it is of interest to be able to partition the input space directly to obtain regions which correspond arbitrarily closely to a ZML distribution. Hence, the next step in the process of symbolization is to determine a direct partitioning algorithm. We describe a method for doing this below.

One possible approach which we term the CDF-ZML method, is to partition the input space into a set with probabilistic characteristics $\left\{{\hat{p}}_{U}\left(\pi_{r}\right)\right\},$ using kernel density estimation [73] or a direct cdf estimation algorithm [74]. From the cdf it is possible to directly partition the input space according to $\left\{{\hat{p}}_{U}\left(\pi_{r}\right)\right\}$.

Using a simple brute force approach, let $\hat{F}\left(u\right)$ be the estimated cdf of the input space, then stepping through the range of the cdf, for each $u_{j},$ find $\hat{F}\left(u_{j}\right).$.

Subsequently we define

(A11) $$\tilde{p}\left(u_{j}\right)=\hat{F}\left(u_{j}\right)-{\hat{F}}_{U}\left(\pi_{r}\right)$$

where $\left\{{\hat{F}}_{U}\left(\pi_{r}\right)\right\}$ is the cdf of the corresponding ZML distribution $\left\{{\hat{p}}_{U}\left(\pi_{r}\right)\right\},$ and hence the set $\left\{\tilde{p}\left(u_{\pi }\right)\right\}$ can be readily determined.

Therefore, this is a simple algorithm which can be used to partition an input space, enabling a memoryless symbolization of an input sequence, where the distribution is constrained to be characterized by a defined Zipf–Mandelbrot–Li distribution.

## Appendix B. Derivation of Intelligibility Maximization (MaxIntel) Algorithm

Language sequences are known to be rich in their expressive capability and can be defined in terms of various statistical properties. Another aspect of language is that it has features of intelligibility, which enable the input to have greater likelihood of recognition under conditions of noise.

Here we present an algorithm which seeks to maximize the intelligibility of a sequence through an appropriate partitioning scheme. The idea is that we place the partitions in such a way that the partitions nearest to each other have the least probability of occurring.

We proceed with the derivation of the algorithm as follows. For a sequence of symbols $\left\{s_{\tau }\right\}$ with probabilities $\left\{\tilde{p}\left(\tau \right)\right\},$ for any given symbol $s_{\tau }$ with probability $\tilde{p}\left(\tau \right),$ then the subsequent partition is defined to correspond to a symbol $s_{\omega }$ where the symbol index ω is determined as:

(A12) $$\omega =\arg\max_{\tau }\chi \left(\tau ;\theta \right)$$

Then an intelligibility measure χ(τ;θ) is defined as

(A13) $$\begin{array}{cc} \chi (\tau ;\theta ) & =\frac{1}{M}\sum_{\tau }^{M}d\left(\tau \right) \end{array}$$

(A14) $$\begin{array}{cc} d(\tau ) & =\alpha \left(\tau \right){\left(\tilde{p}\left(\tau \right)-\tilde{p}\left(\tau -1\right)\right)}^{2} \end{array}$$

(A15) $$\begin{array}{cc} \alpha \left(\tau \right) & =g\left(\tilde{p}\left(\tau \right)\right) \end{array}$$

where $\tilde{p}\left(\tau \right)$ is the set of probabilities of all symbolic elements.

Next, we introduce a scaling factor $g\left(\tilde{p}\left(\tau \right)\right)$ which can be defined in terms of measures such as Jeffreys entropy or relative entropy.

It is necessary to normalize the probability differences. Hence, a simplified probability normalization we choose is

(A16) $$g\left(\tilde{p}\left(\tau \right)\right)=\frac{1}{2}{\left(\tilde{p}\left(\tau \right)+\tilde{p}\left(\tau -1\right)\right)}^{2}$$

which ensures that the probability differences will be normalized by the mean value of the probability bounds.

This allows us to then derive a symbolization algorithm by an ordering method which maximizes intelligibility. We proceed to determine this using the following method.

Let *G* be a connected graph of the probability indices for probabilities $\left\{\tilde{p}\left(\tau \right)\right\}.$ A symbolization algorithm can order the probabilistic regions of an input space in any sequence, and hence is unordered. The Wiener index W(G) is the sum of the distances between all unordered pairs of distinct vertices [75,76] and hence

(A17) $$W\left(G\right)=\sum_{\left\{\tau ,\pi \right\}\subseteq V\left(G\right)}d_{G}\left(\tau ,\pi_{r}\right)$$

where $d_{G}\left(\tau -\pi_{r}\right)$ is distance between vertices. In the present discussion, we consider unimodal probability distributions and hence their corresponding indices. However, this approach can be readily extended to multimodal systems in which case a natural extension to vertices is provided. For our purposes, we consider the unimodal case and hence restrict our discussion to indices in a linear probability distribution $\left\{\tilde{p}\left(\tau \right)\right\}.$ τ and $\pi_{r},$ that is, the minimum number of edges on a $\left(\tau ,\pi_{r}\right)$-path in G.

Now, since the distance is measured in terms of probabilities, we define

(A18) $$d_{G}\left(\tau ,\pi \right)=\alpha \left(\tau ,\pi \right){\left(\tilde{p}\left(\tau \right)-\tilde{p}\left(\pi \right)\right)}^{2}$$

where V(G) is the vertex set and $n\left(G\right)=\left|V(G)\right|$ is the order of G. The maximum intelligibility $\chi_{m}\left(G\right)$ can be defined as a function of the eccentricity $e_{G}\left(\tau \right)$ of a vertex τ which is the distance of a vertex a maximal distance from τ, hence

(A19) $$\begin{array}{cc} \chi_{m}\left(G;\theta \right) & =\frac{1}{M-1}\sum_{\tau }^{M-1}e_{G}\left(\tau ,\theta \right) \end{array}$$

(A20) $$\begin{array}{cc} e_{G}\left(\tau ,\theta \right) & =\max_{\pi_{r}\subset V\left(G\right)}d_{G}\left(\tau ,\pi_{r}\right) \end{array}$$

and

(A21) $$\theta \left(\tau \right)=\arg\max_{\pi_{r}\subset V\left(G\right)}d_{G}\left(\tau ,\pi_{r}\right)$$

Consider the incremental change in intelligibility $\chi_{m}\left(G;\theta_{z}\right)$ for a probability set corresponding to the ZML model $\left\{p_{z}\left(r\right)\right\}$ defined by (A7)–(A9), of size M=k, $p_{z}\left(r\right)<p_{z}\left(r+1\right)$ ∀r, we have, for $k=2,\theta_{z}\left(1;M\right)=2,$ the index of the maximal distance between vertices *i* and *r* is given by

(A22) $$\theta_{z}\left(i,r;M\right)=\arg\max_{k}\left[d_{z}\left(i,k\right)\right]\;r=2,\ldots ,M;i=r-1$$

where we define

(A23) $$d_{Z}\left(\tau ,\pi \right)=\alpha \left(\tau ,\pi \right){\left(\tilde{p}\left(\tau \right)-\tilde{p_{z}}\left(\pi \right)\right)}^{2}$$

Then

(A24) $$\theta_{z}\left(1,r=k;M\right)=M,\;k=2$$

Furthermore, hence

(A25) $$n_{z}\left(G;k\right)=\left[1,M,2,u_{k}\right]$$

where $u_{k}$ is the remainder ordered set. Then, defining the maximal probabilistic distance between vertices τ and π in an ordered set $\left\{\tilde{p}\left(\tau \right)\right\}$ of size *M* as

(A26) $${\hat{d}}_{Z}\left(\tau ,\pi ;M\right)=\alpha \left(\tau ,\pi \right){\left(\tilde{p}\left(\theta_{z}\right)-\tilde{p_{z}}\left(\pi \right)\right)}^{2}$$

Then we have for τ=1,π=2

(A27) $$\begin{array}{cc} \theta_{z}\left(1,2;M\right) & =\arg\max_{k}\left[d_{z}\left(k,k+1\right)\right]\\ \; & =\arg\max_{k}\left\{{\hat{d}}_{z}\left(k,M-j;M\right)\right\}\;j=0,\ldots ,M-1\\ \; & =M \end{array}$$

and since we have populated $\tilde{p}\left(\tau =2\right)\Rightarrow$ i=M, then we have for τ=k,π=k+1

(A28) $$\begin{array}{cc} \theta_{z}\left(k,k+1;M\right) & =\arg\max_{k}\left[d_{z}\left(k,k+1\right)\right]\\ \; & =\arg\max_{k}\left\{{\hat{d}}_{z}\left(k,M-j;M\right)\right\}\;j=k-1,\ldots ,M-\left(k-2\right)/2\\ \; & =\max\left(M-j\right)\;(k\;\mathrm{even})\; \end{array}$$

and by induction, for i=k+1,r=k+2

(A29) $$\begin{array}{cc} \theta_{z}\left(k,r=k+1;M\right) & =\arg\max_{k}\left[d_{z}\left(k,k+1\right)\right]\\ \; & =\arg\max_{k}\left\{{\hat{d}}_{z}\left(k,M-j;M\right)\right\}\;j=k-1,\ldots ,M-\left(k-1\right)/2\\ \; & =\min\left(M-j\right)\;(k\;\mathrm{odd})\; \end{array}$$

and hence $n_{z}\left(G\right)$ can be obtained. Hence, an algorithm which optimizes this intelligibility measure for unigrams is given by

(A30) $$\begin{array}{cc} \chi_{e}\left(2k\right) & =p_{z}\left(k\right)\;k=0,\ldots ,M/2\\ \;\chi_{o}\left(2k-1\right) & =p_{z}\left(M-k\right)\;k=1,\ldots ,M/2\; \end{array}$$

Hence, the resulting algorithm permits the partitioning of the input space into regions which will ensure maximal intelligibility by ensuring the distance between similarly probable events is as large as possible.

## Appendix C. Derivation of LCEM Symbolization Algorithm

The EM algorithm is a well known method of clustering data. Here we propose a probabilistic constrained EM algorithm which seeks to provide synthetic primitives which conform to a Zipfian–Mandelbrot–Li distribution corresponding to the expected distribution properties of a language alphabet.

Consider an iid d-dimensional random vector $x\in R^{d}$ which can be approximated by a multivariate mixture model. This can be described by

(A31) $${\tilde{p}}_{M}\left(x_{i}|\theta \right)=\sum_{k=1}^{K}\alpha_{k}{\tilde{p}}_{k}\left(x_{i}|z_{i}=k,\theta_{k}\right)$$

where $z_{i}$ is a latent, random K-dimensional indicator vector identifying the mixture component generating $x_{i}$ where

(A32) $$1_{\left\{z_{i}=k\right\}}\;=\left\{\begin{array}{cc} 1 & \mathrm{if}\;z_{i}=k\\ 0 & \mathrm{otherwise} \end{array}\right.$$

For a Gaussian mixture distribution, $\left\{\alpha_{k}\right\}$ is the set of mixing coefficients satisfying $\alpha_{k}\geq 0$ and $\sum_{k=1}^{K}\alpha_{k}=1$ with mixture components are defined as

(A33) $${\tilde{p}}_{k}\left(x_{i}|\theta_{k}\right)∼N\left(x_{i}|\theta_{k}\right)$$

with mean values $\mu \in R^{d}$ and symmetric, positive definite covariance matrix $\Sigma \in R^{dxd}$ and $\theta_{i}=\left[\mu_{i},\Sigma_{i}\right].$ Hence ${\tilde{p}}_{k}\left(x_{i}|\theta_{k}\right)$ can be defined in terms of the d-dimensional multivariate normal distribution, where

(A34) $${\tilde{p}}_{k}\left(x_{i}|\theta_{k}\right)=\frac{1}{\sqrt{\left|\Sigma_{k}\right|{\left(2\pi \right)}^{d}}}\exp\left(-\frac{1}{2}{\left(x_{i}-\mu_{k}\right)}^{T}\Sigma_{k}^{-1}\left(x_{i}-\mu_{k}\right)\right)$$

Note that the components can be any parametric density function which need not have the same functional form. To fit a mixture model, we may define the likelihood function

(A35) $$\begin{array}{cc} l\left(\theta \right) & =\sum_{i=1}^{N}\log{\tilde{p}}_{M}\left(x_{i}|\theta \right) \end{array}$$

(A36) $$\begin{array}{cc} \; & =\sum_{i=1}^{N}\log\left(\sum_{k=1}^{K}\alpha_{k}{\tilde{p}}_{k}\left(x_{i}|z_{i}=k,\theta_{k}\right)\right); \end{array}$$

however, finding a maximum likelihood estimate of θ may be difficult. In fact, due to the hidden variables $\left\{z_{i}\right\},$ there may be no closed form solution to determine the parameters θ. Hence, a convenient solution to this problem is the EM algorithm, where although maximizing $l\left(\theta \right)$ might be difficult or impossible, this approach provides an iterative method of repeatedly constructing a lower-bound on $l\left(\theta \right)$ and then optimizing the lower bound using Jensen’s inequality. Hence, this approach performs a local maximization of the log likelihood.

The E-step of the EM algorithm computes the membership weight of data point $x_{i}$ in component *k* with parameters $\theta_{k},$ for i∈[1,N] data points, is defined as

(A37) $$w_{ik}=\frac{\alpha_{k}{\tilde{p}}_{k}\left(x_{i}|z_{i}=k,\theta_{k}\right)}{\sum_{m=1}^{K}\alpha_{m}{\tilde{p}}_{m}\left(x_{i}|z_{m}=k,\theta_{m}\right)}\;k=1,\ldots ,K\;$$

Having obtained the membership weights, the M-step is used to calculate a new set of parameter values. The sum of the membership weights calculated in (A37) can be used to determine the number of points contributing to each component can be now determined as

(A38) $$\alpha_{k}\left(t+1\right)=\frac{N_{k}}{N}\;k=1,\ldots ,K\;$$

where

(A39) $$N_{k}=\sum_{i=1}^{N}w_{ik}$$

Hence, new estimates of the parameters can be obtained as follows. For the Gaussian mixture model, we have

(A40) $$\mu_{k}\left(t+1\right)=\left(\frac{1}{N_{k}}\right)\sum_{i=1ik}^{N}x_{i}w_{ik}\;k=1,\ldots ,K$$

and the updated estimate for the covariances is

(A41) $$\Sigma_{k}\left(t+1\right)=\left(\frac{1}{N_{k}}\right)\sum_{i=1ik}^{N}w_{ik}\left(x_{i}-\mu_{k}\left(t+1\right)\right){\left(x_{i}-\mu_{k}\left(t+1\right)\right)}^{T}$$

Now, a support region can be placed on each mixture to provide a set of probabilities. Hence, for a Gaussian mixture model with adaptive support regions, we introduce the model, parametrized as

(A42) $${\tilde{p}}_{M}\left(x|\varphi \right)=\sum_{k=1}^{K}\alpha_{k}{\tilde{p}}_{k}\left(x_{i}|z_{i}=k,\varphi_{k}\right)$$

where $\varphi_{i}=\left[\mu_{i},{\hat{\mu }}_{i},\Sigma_{i},{\hat{\Sigma }}_{i}\right],$ and a probabilistic bound is associated with each component and is defined by the hyper-volume with edges $\left\{\alpha_{i}\right\},\left\{\mu_{i}\pm {\hat{\mu }}_{i}\right\},\left\{\Sigma_{i}+{\hat{\Sigma }}_{i}\right\}$ resulting in probabilities

(A43) $$p\left(x_{k}|\varphi_{k}\right)=\overset{\mu_{j}+{\hat{\mu }}_{j}}{\underset{\mu_{j}-{\hat{\mu }}_{j}k}{\int \ldots \int }}{\tilde{p}}_{k}\left(x_{k}|z_{k}=1,\varphi_{k}\right)\;j=1,\ldots ,d$$

which can be computed using adaptive quadrature transformations for bivariate and trivariate distributions developed by Drezner and Genz [77,78,79] and for higher dimensions a Genz-Bretz quasi-Monte Carlo integration can be used [80,81].

Now, by assigning each data point to a particular region, either fully or in part, coverage is obtained for the full input space, while conveniently developing a probability that can be assigned to the symbol represented by data points appearing in this space. This overcomes the need for a full tessellation approach adopted previously, since the full input space is implicit in the model. The probabilities can be readily assigned in the latter case by various approaches such as a Bayesian model, a winner-take-all model, or simply by direct use of the mixture weights and the derived probabilities. The choice of which approach to use can be anticipated to be determined by the application.

Here, the proposed linguistic constrained EM (LCEM) algorithm efficiently extends (A37)–(A41) to determine the sorted probabilities associated with each cluster, aligned to a theoretical probability distribution defined by (A1)–(A9), where:

(A44) $$e_{H}\left(x|M,\varphi \right)=H_{z}\left(M\right)-H_{0}\left(x|\varphi \right)$$

where:

(A45) $$H_{0}\left(x|\varphi \right)=-\sum_{i=1}^{M}p_{s}\left(x_{i}|\varphi_{i}\right)\log_{2}p_{s}\left(x_{i}|\varphi_{i}\right)$$

and the probabilities $p_{s}\left(x_{i}|\varphi_{i}\right)$ are the rank ordered probabilities

(A46) $$p_{s}\left(x_{i}|\varphi_{i}\right)=\overset{M}{\underset{k=1}{⋂}}p\left(x_{k}|\varphi_{k}\right)$$

Hence, we introduce constrained EM update equations such that for the Gaussian mixture model, we have

(A47) $${\bar{\mu }}_{k}\left(t+1\right)=\left(\frac{1}{N_{k}}\right)\sum_{i=1ik}^{N}x_{i}w_{ik}\;$$

where

(A48) $$\begin{array}{c} \mu_{k}\left(t+1\right)=\left\{\begin{array}{cc} {\bar{\mu }}_{k}\left(t+1\right) & \mathrm{if}\;t<N_{b}\\ {\bar{\mu }}_{k}\left(t+1\right)+\eta \left(\varsigma ,t\right)e\left(x|M,\varphi \right) & \mathrm{otherwise} \end{array}\right. \end{array}$$

and

(A49) $${\bar{\Sigma }}_{k}\left(t+1\right)=\left(\frac{1}{N_{k}}\right)\sum_{i=1ik}^{N}w_{ik}\left(x_{i}-\mu_{k}\left(t+1\right)\right){\left(x_{i}-\mu_{k}\left(t+1\right)\right)}^{T}$$

with

(A50) $$\begin{array}{c} \Sigma_{k}\left(t+1\right)=\left\{\begin{array}{cc} {\bar{\Sigma }}_{k}\left(t+1\right) & \mathrm{if}\;t<N_{b}\\ {\bar{\Sigma }}_{k}\left(t+1\right)+\eta \left(\varsigma ,t\right)e\left(x|M,\varphi \right) & \mathrm{otherwise} \end{array}\right. \end{array}$$

where $\eta \left(\varsigma ,t\right)$ is an adaptive learning rate defined as

$$\eta \left(\varsigma ,t\right)=\left\{\begin{array}{cc} \epsilon & \mathrm{if}\;t<N_{L}\\ \epsilon \exp\left(\frac{-\rho t}{N_{r}}\right) & \mathrm{otherwise} \end{array}\right.$$

with learning rate ϵ, decay constant ρ, adaptive learning delay $N_{L}$ over $t=1,\ldots ,N_{r}.$

In the LCEM algorithm, the clusters are initialized over $N_{b}$ points, and then the entropic error is minimized progressively as a constraint by adapting the mean and variance of each cluster. In this manner, a new weighting for each cluster is obtained which is a function of the likelihood and the entropic error. This continues until the cluster probabilities converge as indicated by the entropy, or other some other criterion has been met, such as time to converge.

The proposed algorithm is suitable for an online approach where not all of the data are available at once. The LCEM algorithm introduces a novel constraint of seeking to ensure the symbolization process outputs symbols which conform to a Zipf–Mandelbrot–Li distribution. This approach can be extended to introduce further constraints which would improve the expected performance in terms of linguistic feature characterization.
